# A deep-learning pipeline to diagnose pediatric intussusception and assess severity during ultrasound scanning: a multicenter retrospective-prospective study

**DOI:** 10.1038/s41746-023-00930-8

**Published:** 2023-09-30

**Authors:** Yuanyuan Pei, Guijuan Wang, Haiwei Cao, Shuanglan Jiang, Dan Wang, Haiyu Wang, Hongying Wang, Hongkui Yu

**Affiliations:** 1 Provincial Key Laboratory of Research in Structure Birth Defect Disease and Department of Pediatric Surgery, Guangzhou Women and Children’s Medical Center, Guangzhou Medical University, Guangdong Provincial Clinical Research Center for Child Health, Guangzhou, China; 2 School of Computer Science, South China Normal University, Guangzhou, China; 3 Ultrasonic Department, Kaifeng Children’s Hospital, Kaifeng, China; 4 Ultrasonic Department, Dongguan Children’s Hospital, Dongguan, China; 5 Ultrasonic Department, Children’s Hospital Affiliated to Zhengzhou University, Zhengzhou, China; 6 Department of Ultrasonography, Guangzhou Women and Children’s Medical Center, Guangzhou Medical University, Guangzhou, China; 7 Department of Ultrasonography, Shenzhen Baoan Women’s and Children’s Hospital, Jinan University, Shenzhen, China

**Keywords:** Paediatric research, Intestinal diseases

## Abstract

Ileocolic intussusception is one of the common acute abdomens in children and is first diagnosed urgently using ultrasound. Manual diagnosis requires extensive experience and skill, and identifying surgical indications in assessing the disease severity is more challenging. We aimed to develop a real-time lesion visualization deep-learning pipeline to solve this problem. This multicenter retrospective-prospective study used 14,085 images in 8736 consecutive patients (median age, eight months) with ileocolic intussusception who underwent ultrasound at six hospitals to train, validate, and test the deep-learning pipeline. Subsequently, the algorithm was validated in an internal image test set and an external video dataset. Furthermore, the performances of junior, intermediate, senior, and junior sonographers with AI-assistance were prospectively compared in 242 volunteers using the DeLong test. This tool recognized 1,086 images with three ileocolic intussusception signs with an average of the area under the receiver operating characteristic curve (average-AUC) of 0.972. It diagnosed 184 patients with no intussusception, nonsurgical intussusception, and surgical intussusception in 184 ultrasound videos with an average-AUC of 0.956. In the prospective pilot study using 242 volunteers, junior sonographers’ performances were significantly improved with AI-assistance (average-AUC: 0.966 vs. 0.857, *P* < 0.001; median scanning-time: 9.46 min vs. 3.66 min, *P* < 0.001), which were comparable to those of senior sonographers (average-AUC: 0.966 vs. 0.973, *P* = 0.600). Thus, here, we report that the deep-learning pipeline that guides lesions in real-time and is interpretable during ultrasound scanning could assist sonographers in improving the accuracy and efficiency of diagnosing intussusception and identifying surgical indications.

## Introduction

Intussusception is one of the common acute abdomens in children, with the ileocolic type being the most prevalent, which is usually diagnosed urgently using ultrasound^1^. Nonsurgical enemas administered within 24 h of onset relieve symptoms in approximately 84% of patients^2,3^. Less than one-third of patients present with the classic triad of symptoms (abdominal pain, palpable mass, and blood stained stools), and some pediatric diseases have similar clinical manifestations^4^. Thus, its diagnosis can be easily delayed or missed during the initial emergency visit, delayed treatment can cause sepsis or even hypovolemic shock^5^. Patients with severe symptoms or failed enemas require prompt identification of surgical indications^6,7^.

The sensitivity and specificity of ultrasound for intussusception can achieve 92–100% of diagnosis^8,9^. However, the speed of the ultrasound scan exceeds 25 frames per second (FPS). An ultrasound scan includes thousands of image frames, and only a few frames with clear pathological features are useful for the diagnosis of intussusception and are thus easily missed by the human eye. In addition, noise, distortion, and artifacts degrade the quality of ultrasound images^10^. Therefore, specialized imaging knowledge and skilled sonographers are required to diagnose whether it is intussusception, and the recognition of surgical indications to assess disease severity is more challenging.

Artificial intelligence (AI) has demonstrated general applicability in diagnosing pediatric diseases using medical images^11,12^. Previous studies have also proposed deep-learning (DL) approaches to extract frames with complete pathological features from ultrasound videos for diagnosis^13,14^. AI using images labeled with a priori knowledge to diagnose intussusception has also been reported. However, they did not consider the algorithm’s speed and provide surgical indications^15,16^. Furthermore, intuitively understanding the internal decision-making process of DL is challenging, thereby hindering its translation into clinical practice.

We aim to develop and validate a deep-learning pipeline for real-time navigation of diagnostic planes during ultrasound scanning to identify ileocolic intussusception and provide surgical indications using heterogeneous multicenter datasets of images and videos for retrospective testing and external validation. The tool’s scalability is prospectively evaluated using a real-world clinical dataset. The performance of junior sonographers with AI-assistance is compared with those of junior, intermediate, and senior sonographers. We also attempt to visually interpret the “black box” of DL’s internal decision-making to boost the sonographer’s confidence in the algorithm.

## Results

### Characteristics of patients

**Fig. 1:**
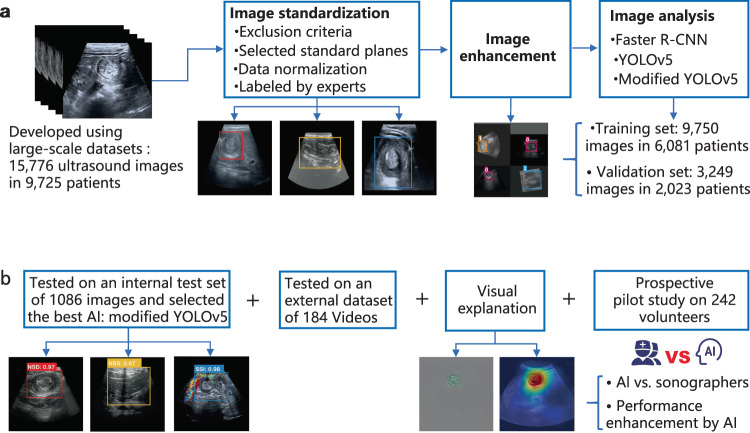
The AI system for diagnosing ileocolic intussusception and providing surgical indications. **a** Model development for the AI system and selecting the best-performing model. The system includes a pipeline consisting of an image normalization module, an image enhancement module, and an image analysis module. **b** Application and evaluation of the AI system.

Figure 1 illustrates the flow diagram of this study. Epidemiological characteristics, medical image features, and final diagnoses for the three data sets are summarized in Table 1. In the retrospective datasets with 14,085 images from 8736 children with ileocolic intussusception, the median age was eight months (range, 3–36), 63.5% were male, 8.4% required surgery, and the classic triad of symptoms was observed in less than one-third of the patients.

**Table 1:** Datasets for training, validation, testing, prospective pilot study, and Characteristics of patients.

Characteristics	A retrospective dataset (images)			An external retrospective validation set (videos)	A prospective dataset (volunteers)
	Training set	Validation set	Test set		
Number of patients	6081	2023	632	184	242
**Final diagnosis** N (%)					
NON	0 (0.0)	0 (0.0)	0 (0.0)	28 (15.2)	43 (17.7)
NSI	5584 (91.8)	1880 (92.9)	551 (87.2)	137 (74.5)	178 (73.6)
SIP	497 (8.2)	143 (7.1)	81 (12.8)	19 (10.3)	21 (8.7)
**Number of labeled images**	9750	3249	1086		
**Labeled ultrasound image categories** N (%) *					
NSD	7628 (78.2)	2678 (82.4)	820 (75.5)		
NSS	1297 (13.3)	336 (10.3)	146 (13.4)		
SSI	825 (8.5)	235 (7.2)	120 (11.0)		
**Patients’ characteristics**					
**Age** Median age (range)					
Month	8 (3–36)	8 (3–36)	9 (3–36)	8 (3–36)	9 (3–36)
**Sex** *N* (%)					
Male	3899 (64.1)	1317 (65.1)	412 (65.2)	112 (60.9)	151 (62.4)
Female	2182 (35.9)	706 (34.9)	220 (34.8)	72 (39.1)	91 (37.6)
**Patients’ clinical symptoms** *N* (%)					
Abdominal pain	5304 (87.2)	1689 (83.5)	579 (91.6)	156 (84.8)	216 (89.3)
Vomiting	4975 (81.8)	1551 (76.7)	523 (82.7)	132 (71.7)	180 (74.4)
Currant jelly stool	2112 (34.7)	738 (36.5)	241 (38.1)	74 (40.2)	93 (38.4)
Abdominal mass	1693 (27.8)	559 (27.6)	156 (24.7)	40 (21.7)	55 (22.7)
Rectal examination with blood	1529 (25.1)	452 (22.3)	137 (21.7)	38 (20.7)	58 (24.0)
Temperature >38.5°	734 (12.1)	221 (10.9)	81 (12.8)	14(7.6)	22 (9.1)
Irritability, paroxysmal crying	3729 (61.3)	1289 (63.7)	361 (57.1)	94 (51.1)	132 (54.5)
Drowsiness	1021 (16.8)	293 (14.5)	87 (13.8)	22 (12.0)	34 (14.0)

*N* = Number of patients, images or videos. Data are presented as *n* (%) or median (range). *Percentages may not add to exactly 100% because of rounding. *NON* Normal or not intussusception patients, *NSI* Nonsurgical intussusception patients, *SIP* Surgical intussusception patients, *NSD* Nonsurgical doughnut sign, *NSS* Nonsurgical sleeve sign, *SSI* Surgical sign.

### Training, validating, and selecting the optimal deep-learning model

The three AI (Faster RCNN, YOLOv5, and Modified YOLOv5) were trained using a training set of 9,750 images from 6,081 patients and a validation set of 3,249 images from 2,023 patients. Subsequently, the average-AUC and median FTP of the modified YOLOv5 were comparable with those of Faster R-CNN (average-AUC: 0.976 [95% CI, 0.967–0.983] vs. 0.793 [95% CI, 0.782–0.805], *P* < 0.001; median FTP: 102 [range 93–109] vs. 25 [range, 20–31], *P* < 0.001) and YOLOv5 (average-AUC: 0.976 [95% CI, 0.967–0.983] vs. 0.981 [95% CI, 0.972–0.986], *P* = 0.698; median FTP of 102 [range, 93–109] vs. 63 [range 57–59], *P* < 0.001) in the internal test set of 1,086 images. Thus, we selected the best-performing modified version of YOLOv5.

### Testing on the internal retrospective image test set

**Fig. 2:**
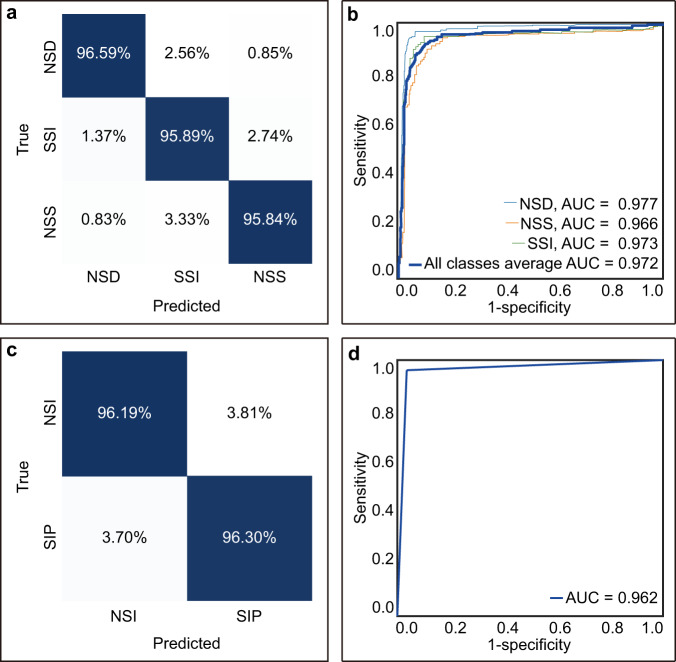
Prediction results of the deep-learning system on the internal test set of 1,086 ultrasound images in 632 patients. **a** Normalized confusion matrix of images. **b** AUCs of images. **c** Normalized confusion matrix of patients. **d** AUC of patients.

The deep-learning system was tested on the internal test set containing 1,086 images from 632 patients. Three types of images with nonsurgical doughnut, nonsurgical sleeve, and surgical signs were identified with an AUC of 0.977 (95% CI, 0.968–0.986), 0.966 (95% CI, 0.949–0.983), and 0.973 (95% CI, 0.955–0.992), respectively, with a confusion matrix and an average-AUC of 0.972 (95% CI, 0.936–1.000). The corresponding AUC for diagnosing these patients with ileocolic intussusception requiring surgery was 0.962 (95% CI, 0.940–0.985) (Fig. 2 and Table 2).

**Table 2:** Performance of the deep-learning system on the internal test set of 1086 ultrasound images in 632 patients.

Images	Accuracy (95%CI)	Sensitivity (95%CI)	Specificity (95%CI)	AUC (95%CI)	FK (95%CI)	Average-AUC (95%CI)	FPS Median (range)
NSD	0.971 (0.971–0.972)	0.966 (0.953–0.978)	0.989 (0.976–1.000)	0.977 (0.968–0.986)	0.925 (0.899–0.951)	0.972 (0.954–0.994)	102 (93–109)
NSS	0.971 (0.971–0.972)	0.959 (0.927–0.991)	0.973 (0.963–0.984)	0.966 (0.949–0.983)	0.884 (0.844–0.924)		
SSI	0.985 (0.985–0.985)	0.958 (0.923–0.994)	0.989 (0.982–0.995)	0.973 (0.955–0.992)	0.927 (0.891–0.962)		

*AUC* The area under the receiver operating characteristic curve, *Average-AUC* the arithmetic mean of the *AUC* for each class, *FK* Fleiss’ Kappa, *FPS* Frames per second.

### Model generalisation to an external retrospective video dataset

We added the “Con-best.py” program to the YOLOv5 model to select the optimal standard diagnostic plane with the highest Confidence in each video. Subsequently, the AI system was tested using 184 ultrasound videos from 184 patients (including cases with no intussusception, nonsurgical intussusception, and surgical intussusception) with an AUC of 0.958 (95% CI, 0.909–1.000), 0.953 (95% CI, 0.919–0.988), and 0.956 (95% CI, 0.902–1.000), respectively, with an average-AUC of 0.956 (95% CI, 0.961–0.991) and a median FPS of 91 (range, 83–101) (Table 3). The model not detecting the nonsurgical doughnut, nonsurgical sleeve, or surgical signs in a video indicated that the patient did not have ileocolic intussusception.

**Table 3:** Performance of the deep-learning system on the external retrospective 184 videos in 184 patients.

Patients	Accuracy (95%CI)	Sensitivity (95%CI)	Specificity (95%CI)	AUC (95%CI)	FK (95%CI)	Average-AUC (95%CI)	FPS Median (range)
NON	0.978 (0.978–0.978)	0.929 (0.833–1.000)	0.987 (0.970–1.000)	0.958 (0.909–1.000)	0.916 (0.834–0.997)	0.956 (0.0.913–0.998)	91(83–101)
NSI	0.951 (0.951–0.952)	0.949 (0.912–0.986)	0.957 (0.900–1.000)	0.953 (0.919–0.988)	0.876 (0.797–0.955)		
SIP	0.962 (0.962–0.962)	0.947 (0.847–1.000)	0.964 (0.935–0.992)	0.956 (0.902–1.000)	0.816 (0.684–0.948)		

### Visual interpretation of deep-learning internal decision-making

**Fig. 3:**
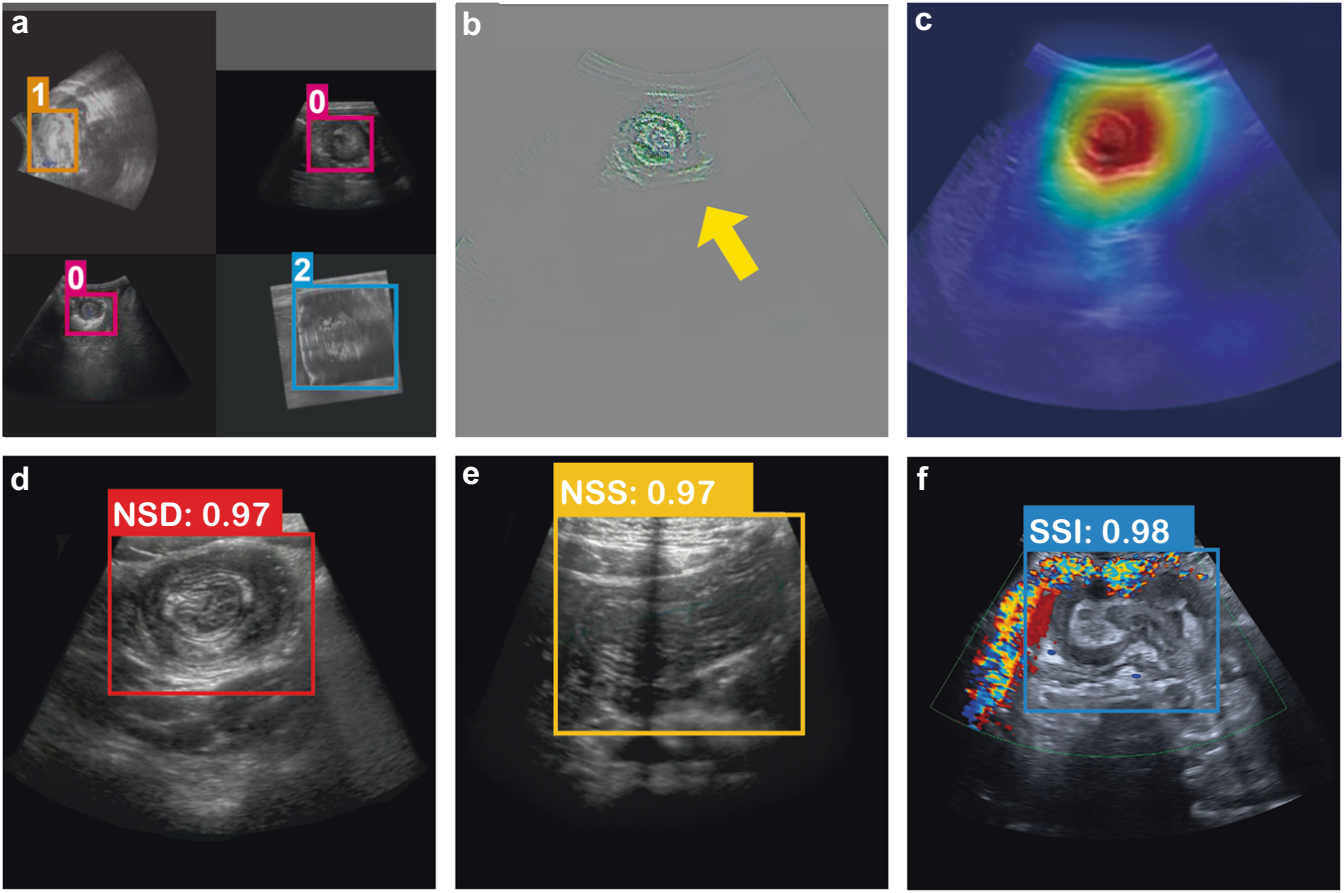
Visual interpretation of deep-learning internal decision-making. **a** The result of “Mosaic” image enhancement. The “0,” “1,” and “2” represent the three types of images. The anchor boxes represent the labeling of the lesion region. **b** The feature map. **c** Heat map was generated by extracting the feature map from the last convolutional layer, and the red and yellow regions indicate the nonsurgical donut signs identified by the AI system. **d, e and f** The AI system automatically identified the images as NSD, NSS, and SSI and labeled the lesion areas with Confidence of 0.97, 0.97, and 0.98, respectively. NSD Nonsurgical doughnut sign, NSS Nonsurgical sleeve sign, SSI Surgical sign.

Four images were cropped at any angle, and their brightness and contrast were adjusted. Then they were stitched into a “Mosaic” enhanced image (Fig. 3a). The learning effect of each convolutional layer can be explained using the visualized feature maps. Here, the last convolutional layer of YOLOv5 was chosen to map the features to the range of 0–255 and convert them into images. The binary grayscale image revealed that the convolutional layer learned to recognize the doughnut sign (Fig. 3b). A heat map, which visualizes the activation of convolutional layer features, further aided in determining whether the model can correctly identify image features. A heat map was created by extracting the activation value from the last convolutional layer of YOLOv5 and multiplying it with the average gradient feature map value. The red and yellow regions in Fig. 3c signify the model’s identification of the lesion area. The AI system automatically identified the images as a nonsurgical doughnut, nonsurgical sleeve, and surgical signs and labeled the corresponding lesion areas with a Confidence of 0.97, 0.97, and 0.98, respectively. The values in Fig. 3d–f represent the Confidence, calculated using Eq. 1. In this equation, P (object) was assigned 1 when the category was accurately predicted and 0 otherwise.

1 $${Confidence}=P({object})* {IoU}\left({truth}- > {pred}\right)$$

### Generalizing the model to real-world ultrasound diagnostic scenarios and comparing the performances of four groups of sonographers

**Fig. 4:**
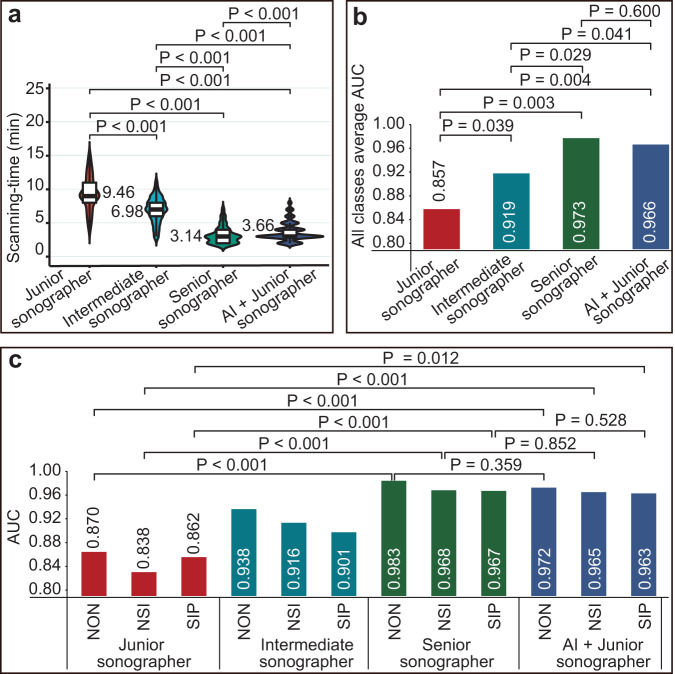
The performance of the four groups of sonographers was compared among 242 volunteers. **a** Comparison of the scanning-time among the four groups of sonographers. The central line marks the median scanning-time value, and the bounds of the box mark the first and third quartiles. **b** The average AUCs of the four groups of sonographers for the diagnosis of the three categories of patients were compared using R (version 4.1.1, R Core Team, 2021) programming, calling the “pROC” package and the “DeLong” test. **c** The AUCs of the four groups of sonographers for the diagnosis of the three categories of patients were compared. *P* = *P* value.

We connected this tool to ultrasound machines to assess the performance of the deep-learning pipeline in a real-world ultrasound scanning scenario, and the four sonographer groups with different skill levels conducted replicate ultrasound examinations on each of the 242 patients suspected to have intussusception. Sonographers with equal skill levels from the same department were assigned to the same group to ensure consistency among the observers in each group. The four sonographer groups were as follows: two senior sonographers (13 and 15 years of experience); two intermediate sonographers (6 and 7 years of experience); two junior sonographers (2 years of experience); and two junior sonographers with AI-assistance. The diagnostic results of both sonographers within each group were averaged, with average AUCs of 0.973 (95% CI, 0.938–1.000), 0.919 (95% CI, 0.862–0.979), 0.857 (95% CI, 0.808–0.912), and 0.966 (95% CI, 0.923–0.999), along with median scanning times (in minutes) of 3.14 (interquartile range [IQR], 2.02–4.13), 6.98 ([IQR], 5.89–8.03), 9.46 ([IQR], 7.91–11.17), and 3.66 ([IQR], 2.91–4.21), respectively (Fig. 4, Table 4, and Supplementary Fig. 1).

**Table 4:** Performance of the four groups of sonographers in the diagnosis of 242 volunteers.

Observers	Patients	Accuracy (95%CI)	Sensitivity (95%CI)	Specificity (95%CI)	AUC (95%CI)	FK (95%CI)	Average -AUC	ST (Median [IQR])
Junior sonographers	NON	0.921 (0.921–0.922)	0.791 (0.669–0.912)	0.950 (0.919–0.980)	0.870 (0.807–0.934)	0.734 (0.621–0.847)	0.857 (0.802–0.932)	9.46 (7.91–11.17)
	NSI	0.843 (0.842–0.844)	0.848 (0.796–0.901)	0.828 (0.736–0.921)	0.838 (0.785–0.892)	0.626 (0.520–0.733)		
	SIP	0.905 (0.904–0.906)	0.810 (0.642–0.977)	0.914 (0.877–0.951)	0.862 (0.774–0.950)	0.547 (0.386–0.708)		
Intermediate sonographers	NON	0.959 (0.958–0.959)	0.907 (0.820–0.994)	0.970 (0.946–0.994)	0.938 (0.893–0.984)	0.861 (0.777–0.945)	0.919 (0.879–0.959)	6.98 (5.89–8.03)
	NSI	0.913 (0.913–0.914)	0.910 (0.868–0.952)	0.922 (0.856–0.988)	0.916 (0.877–0.955)	0.789 (0.703–0.874)		
	SIP	0.938 (0.938–0.938)	0.857 (0.707–1.000)	0.946 (0.916–0.976)	0.901 (0.823–0.980)	0.672 (0.519–0.826)		
Senior sonographers	NON	0.988 (0.988–0.988)	0.977 (0.932–1.000)	0.990 (0.976–1.000)	0.983 (0.960–1.000)	0.958 (0.911–1.000)	0.973 (0.953–0.993)	3.14 (2.02–4.13)
	NSI	0.967 (0.967–0.967)	0.966 (0.940–0.993)	0.969 (0.926–1.000)	0.968 (0.942–0.993)	0.917 (0.860–0.973)		
	SIP	0.979 (0.979–0.980)	0.952 (0.861–1.000)	0.982 (0.964–0.999)	0.967 (0.920–1.000)	0.878 (0.772–0.983)		
AI + Junior sonographers	NON	0.983 (0.983–0.984)	0.953 (0.891–1.000)	0.990 (0.976–1.000)	0.972 (0.939–1.000)	0.943 (0.889–0.998)	0.966 (0.621–0.847)	3.66 (2.91–4.21)
	NSI	0.963 (0.963–0.963)	0.961 (0.932–0.989)	0.969 (0.926–1.000)	0.965 (0.939–0.991)	0.907 (0.847–0.966)		
	SIP	0.971 (0.971–0.971)	0.952 (0.861–1.000)	0.973 (0.951–0.994)	0.963 (0.915–1.000)	0.835 (0.716–0.954)		

The time requirements are measured with stopwatches and converted to min when statistics. *ST* Scanning-time, *AI* Artificial intelligence, *IQR* Interquartile range.

## Discussion

In this study, we developed a real-time multiobjective detection and tracking deep-learning model using multicenter heterogeneous datasets and ultrasound imaging characteristics to diagnose ileocolic intussusception and provide surgical indications and tested retrospectively and prospectively. We assessed the retrospective and prospective data. This model achieved average AUCs of 0.972 and 0.956 on the internal image test set and the external video dataset, respectively. In the real-world ultrasound diagnostic scenarios in 242 volunteers, the performance of junior sonographers was significantly improved with AI-assistance (average-AUC: 0.966 vs. 0.857, *P* < 0.001; median scanning time: 9.46 min vs. 3.66 min, *P* < 0.001), which surpassed that of intermediate sonographers (average-AUC: 0.966 vs. 0.919, *P* = 0.039; median scanning time: 9.46 min vs. 6.98 min, *P* < 0.001), but was comparable to that of senior sonographers (average-AUC: 0.966 vs. 0.973; *P* = 0.600). Overall, this diagnostic tool can assist sonographers in managing children with ileocolic intussusception.

Accurate and timely diagnosis of ileocolic intussusception and recognizing surgical indications are critical for selecting treatment plans and achieving positive treatment outcomes^1–3,7^. Studies have proposed using deep-learning to diagnose intussusception in children with plain abdominal radiographs^15,16^. However, X-rays are less sensitive and specific for diagnosing intussusception than ultrasound^17^. Our algorithm achieved a higher AUC using three ultrasound datasets. Additionally, this tool processed images at a median FPS of 91 (range, 83–101) during the ultrasound scan. It displayed Confidence values and anchor boxes to guide the sonographer in adjusting the scan position in real-time, thereby enhancing the diagnostic accuracy and efficiency of less experienced sonographers. The examination time of junior sonographers was reduced from 9.46 ([IQR], 7.91–11.17) min to 3.66 min ([IQR], 2.91–4.21), which is particularly valuable because children <age 3 often do not cooperate during ultrasound scans and tend to cry. Furthermore, our algorithm identified surgical indications, facilitating the assessment of disease severity and increasing confidence in selecting the appropriate treatment options. Nevertheless, intussusception is a dynamic disease. Despite the diagnosis of a surgical indication by an experienced sonographer, a few patients whose abdomens were opened did not require partial bowel resection. Therefore, in clinical practice, even with surgical indications, preference is given to conservative, nonsurgical enemas to minimize surgical risk. Surgery is considered only after 1–3 failed enemas, depending on the status of each patient’s surgical indication.

The proposed model is stable and compatible because it was trained using a multicenter and multi-device dataset and tested with image, video, and real-world clinical datasets. YOLOv5 has three sets of multiscale adaptive anchor boxes that can be adapted to deviations in the physical size of images caused by different ultrasound systems^18^. The tool can be easily applied in clinical practice by connecting it to an ultrasound machine, which is particularly helpful for junior and intermediate sonographers. Furthermore, we have added a “Con-best.py” file to YOLOv5, which can select the optimal standard plane with the highest confidence in a postultrasound video. This addition will further improve the accuracy of the sonographer’s diagnosis because even skilled senior sonographers find it challenging to select the standard plane with the highest confidence in a high-speed ultrasound scan.

The AI system detects differential and subtle features of medical images, even beyond the observational ability and comprehension of the clinicians^19,20^. Differences in ultrasound imaging between nonsurgical and surgical intussusception have also been studied^21–27^. Based on the combined evidence, we suggest that a deep-learning pipeline trained using a dataset labeled with a priori knowledge can diagnose intussusception and provide surgical indications.

Our study has some limitations. First, the model was trained and validated using ileocolic intussusception ultrasound datasets and did not involve other types of intussusception, such as ileoileocolic, enteroenteric (including jejunojejunal and ileoileal), and colocolic types. However, over 90% of intussusceptions are ileocolic^3,28^, this limitation affects the generalizability of the model. When used to diagnose all types of intussusception, false negatives may lead to delayed treatment, whereas false positives may result in unnecessary enemas or surgical interventions. Second, the few surgical intussusception samples might have also affected the model’s performance, despite increasing the number of images using image enhancement techniques. Third, the AI system cannot diagnose intussusception in adults due to the adult intestine’s high gas and fat content and the poor quality of ultrasound images, thereby necessitating computed tomography scans or X-rays.

In conclusion, a deep-learning pipeline based on heterogeneous multicenter ultrasound datasets and their imaging features can assist sonographers in diagnosing pediatric ileocolic intussusceptions and provide surgical indications for assessing disease severity. Further training and validation using datasets involving additional types of intussusception and new technologies are needed to enhance the generalizability and performance of the model.

## Methods

### Datasets

The heterogeneous multicenter dataset included a retrospective dataset of images for training and internal testing, a retrospective dataset of videos for external testing, and a prospective dataset of volunteers to compare the performance of junior sonographers with AI-assistance with that of junior, intermediate, and senior sonographers (Fig. 1). After screening, the final eligible data were collected from six hospitals: three regional hospitals affiliated with the Guangzhou Women’s and Children’s Medical Center, namely: the Children Branch (4781 images from 2842 patients, 61 videos from 61 patients, and 242 volunteers), Zengcheng Branch (2360 images from 1409 patients and 27 videos from 27 patients), and Zhujiang New Town Branch (1748 images from 1184 patients and 33 videos from 33 patients); Children’s Hospital of Zhengzhou University (2791 images from 1,651 patients and 25 videos from 25 patients); Kaifeng Children’s Hospital (1361 images from 981 patients and 17 videos from 17 patients); and Dongguan Children’s Hospital (1044 images from 669 patients and 25 videos from 25 patients).

All parents of the volunteers agreed to their children’s participation in the prospective study. In the retrospective dataset, parents were informed in the initial admission form that their children’s clinical data might be used for the study. Subsequently, the data of those whose parents did not object were included. The study was approved by the local ethics committee and institutional review board of each hospital (Guangzhou Women and Children’s Medical Center: [2021] No. 486B01; Children’s Hospital Affiliated to Zhengzhou University: 2022-H-K29; Kaifeng Children’s Hospital: [2021] 127; Dongguan Children’s Hospital: LL2022121501).

### Retrospective image datasets

The initial image dataset included 15,776 images of ileocolic intussusception containing nonsurgical doughnut, nonsurgical sleeve, and surgical signs in 9725 patients aged 3–36 months who underwent ultrasound between January 2017 and December 2021. It also included 1–8 images with typical pathological features in each child from the electronic medical record systems. Inclusion criteria were based on the discharge outcomes of the patients, image quality, and the consensus of three ultrasound experts who reviewed the data (Prof. Haiwei Cao, Prof. Hongkui Yu, and Prof. Hongying Wang with 13, 15, and 21 years of experience, respectively). In total, 1,048 initially incorrectly extracted diagnostic planes from 629 patients were excluded: (1) 264 from 147 patients with incomplete or poor quality pathologic features; (2) 614 from 379 patients whose initial ultrasounds were misdiagnosed as other abdominal conditions, but who were ultimately diagnosed with nonsurgical or surgical intussusception; (3) 143 from 91 patients initially diagnosed with nonsurgical or surgical intussusception using ultrasound, but eventually diagnosed with other abdominal conditions; (4) 27 from 12 patients misdiagnosed as intussusception requiring surgery, but did not need partial bowel resection on opening.

### Retrospective video datasets

The video dataset included patients first seen in the emergency department for acute abdominal conditions between October 2021 and June 2022, and only those the emergency department physicians initially diagnosed with suspected intussusception based on experience and rapid laboratory tests were sent to our ultrasound department for further examination. Patients with other types of intussusception (*n* = 15) and other abdominal diseases (*n* = 7), according to the actual outcome of the patient’s final discharge, were excluded. In addition, poor-quality videos (*n* = 11) were excluded. The final 184 patients with ultrasound diagnoses of no intussusception, nonsurgical ileocolic intussusception, or surgical ileocolic intussusception were selected, with one video selected for each patient.

### Prospective volunteer datasets

Patients who were initially diagnosed with suspected intussusception in the emergency department and referred to our ultrasound department for further examination were recruited as volunteers between April 2023 and May 2023. Based on the patient’s final discharge record, patients with other types of intussusception (*n* = 21) and other acute abdominal diseases (*n* = 27) were not included in the statistical analysis. The final 242 patients, including those with no intussusception, nonsurgical ileocolic intussusception, and surgical ileocolic intussusception, were used to prospectively validate the scalability of this system and compare the performance of the four sonographer groups with different skill levels in real-world scenarios.

### Ultrasound equipment

The ultrasound equipment used was as follows: (1) LOGIQ E10 and E10 R7 (GE Healthcare, United States) with the C2-9-D array probes. (2) EPIQ Elite (Philips Medical System, the Netherlands) with a C10-3v probe. (3) Acuson Sequoia 512 (Siemens Medical Solutions, United States) with a 4C1 vector transducer. (4) Acuson S3000 (Siemens Healthcare, Germany) with an EC9-4 probe. (5) RS85 Prestige (Samsung Medison Co., South Korea) with a C2-8 probe. (6) DC-80 and Resona Version 7.0 (Mindray Medical, China) with the 6C2 probes. (7) Arietta 850 (Esaote, China) with an UST-9123 abdominal probe. (8) Aplio i800 (Toshiba Medical Systems Corp, Tokyo, Japan) with a P7-3 abdominal probe. (9) Edge II (FUJIFILM SonoSite Inc., United States) with a C35x convex array probe. (10) Aplio i900 (Canon Medical Systems, Japan) with a PVT-375BT convex array probe. (11) MyLab X8 (Esaote, Italy) with a C353 convex probe. (12) S50 (SonoScape Medical Co., China) with a C6-2E micro-convex array probe.

### Ultrasound image analysis

**Fig. 5:**
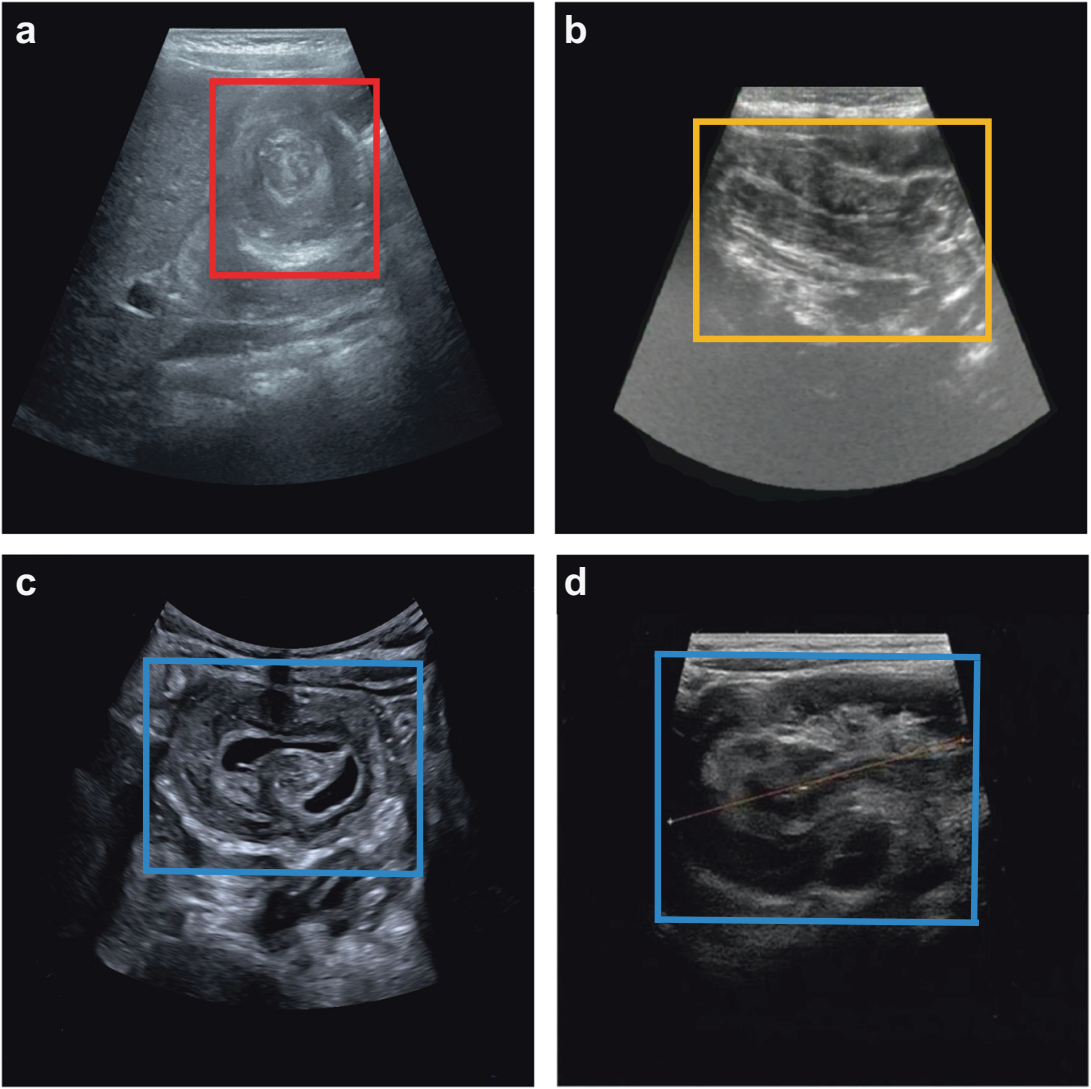
Three types of ileocolic intussusception lesions were labeled by ultrasound experts. **a** NSD Nonsurgical doughnut sign. **b** NSS Nonsurgical sleeve sign. **c, d** SSI Surgical sign.

According to the guidelines for managing intussusception in children^29^, ultrasound imaging of intussusception displays typical features. It appears as a doughnut and a sleeve sign in the transverse and longitudinal view, respectively. These images with clear and complete pathological features are standard planes^8,9^. Compared with temporary intussusception, surgical intussusception presents more coexisting features such as longer intussusception, thicker edematous intestinal wall, larger doughnut diameter, pneumoperitoneum, signs of peritonitis, peritoneal fluid, and “trapped” fluid between the intestinal walls, which may indicate a higher surgical risk (Fig. 5)^21–24^. In this study, In this study, the aforementioned evidence was the ‘gold standard’, while the patient’s final discharge record and the ultrasound expert’s labeling of the images were employed as the ‘silver standard’ for diagnosis.

Two pediatric ultrasound experts (Prof. Haiwei Cao and Prof. Hongkui Yu) selected 14,085 standard planes in 8,736 patients with ileocolic intussusception via consensus. Subsequently, three types of lesions (nonsurgical doughnut, nonsurgical sleeve, and surgical sign) were labeled using their expertise and in conjunction with the patient’s discharge records using an online image labeling and intelligence enhancement tool (Roboflow, https://www.roboflow.com). Due to the fact that some ultrasound images of intussusception are not easily distinguishable into the surgical or nonsurgical types, and there were also some initially extracted standard planes that were incorrect, leading to inconsistencies between the initial diagnostic results and the actual results of the final discharge, which require review in the discharge record and exclusion by the ultrasound experts. In cases of disagreement, a third pediatric ultrasound expert (Prof. Hongying Wang) was consulted.

### AI Model

**Fig. 6:**
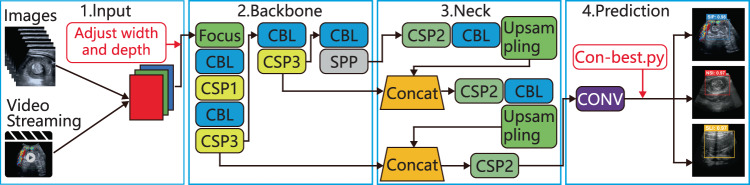
The modified YOLOv5 for diagnosis of pediatric intussusception and providing surgical indications. Its structure consists of a backbone network to extract features, a feature pyramid to obtain multiscale features, a prediction head to generate target predictions, and a loss function to optimize the model.

We modified the YOLOv5 algorithm (https://github.com/ultralytics/yolov5) for real-time “intelligent navigation” of the standard planes to diagnose ileocolic intussusception and provide surgical indications during ultrasound scanning (Fig. 6). Previous studies have also proposed the term “intelligent navigation”^30,31^. Here, “intelligent navigation” refers to the automatic recognition of standard planes during ultrasound scanning. The model displays anchor boxes and Confidence values on the lesions, prompting the sonographer to adjust the position and direction of the scan to capture the optimal standard plane.

The YOLOv5 predicts the boundaries of targets in images, classifies and localizes them using probability, and achieves end-to-end image detection, with the ability to process images at 45–155 FPS^18^. Within the YOLOv5 framework, ‘depth_multiple’ signifies the model’s depth, determining the number of modules (number × depth). Similarly, ‘width_multiple’ represents the model’s width, regulating the count of convolutional channels (number × width). We selected ‘depth_multiple’ = 0.33 and ‘width_multiple’ = 0.50. In this configuration, the network’s depth was reduced by a factor of three, and the number of convolutional channels was reduced by half. This approach enhances image processing speed and decreases reliance on high-end computer hardware configurations.

Data augmentation is used to expand datasets to improve convolutional neural network performance, making models more robust and preventing overfitting^32^. The Roboflow was used to pre-process, normalize and label the three types of lesions in the selected standard planes. Optical features of the image (brightness, hue, and saturation) were adjusted, along with the features geometry of the images. The “Mosaic” enhancement feature of YOLOv5 was used to stitch four images together into a single image. This significantly improves the model’s ability to recognize images with weak features^33^.

The model outputs standard planes with different confidence values during each ultrasound scan. Therefore, we added a “Con-best.py” file to select the standard plane with the highest confidence value from each video after the ultrasound scan.

### Statistical analysis

Normalized confusion matrices were used to depict the classification results of the three types of patients. AUC with 95% confidence intervals (CIs) of the four sonographer groups (junior, intermediate, senior sonographers, and junior sonographers with AI-assistance) and the three AI (Faster R-CNN [https://github.com/rbgirshick/py-faster-rcnn], YOLOv5, and modified YOLOv5) were compared using the DeLong test and “pROC” package. Wilcoxon signed-rank test was used to compare the scanning time of the four observer groups and the median FPS of the three AIs because the non-normality of these data distributions was assessed beforehand using the Kolmogorov–Smirnov test. The performances of the four sonographer groups were evaluated using accuracy, sensitivity, specificity, AUC, and Fleiss’ Kappa. The shortest two-sided 95% CIs were reported for each experiment. Data were analyzed using R statistical software (version 4.1.1, R Core Team, 2021). *P* < 0.05 was considered indicative of a statistically significant difference.

### Supplementary information

Supplementary_Figure

## Data Availability

The ultrasound images and videos are not publicly available by hospital regulations to protect patient privacy. Limited data access is obtainable upon reasonable request by contacting the corresponding author.

## Code availability

The modified YOLOv5 model has been made publicly available at Github (https://github.com/yuanyuanpei7/pediatric-intussusception).
