# Supplementary material for: A deep-learning pipeline to diagnose pediatric intussusception and assess severity during ultrasound scanning: a multicenter retrospective-prospective study
Source: NPJ Digit Med. 2023 Sep 30;6:182. doi: 10.1038/s41746-023-00930-8 (PMC10541898; doi:10.1038/s41746-023-00930-8)
Supplement: Supplementary file 1 — Supplementary_Figure [file 41746_2023_930_MOESM1_ESM.pdf]

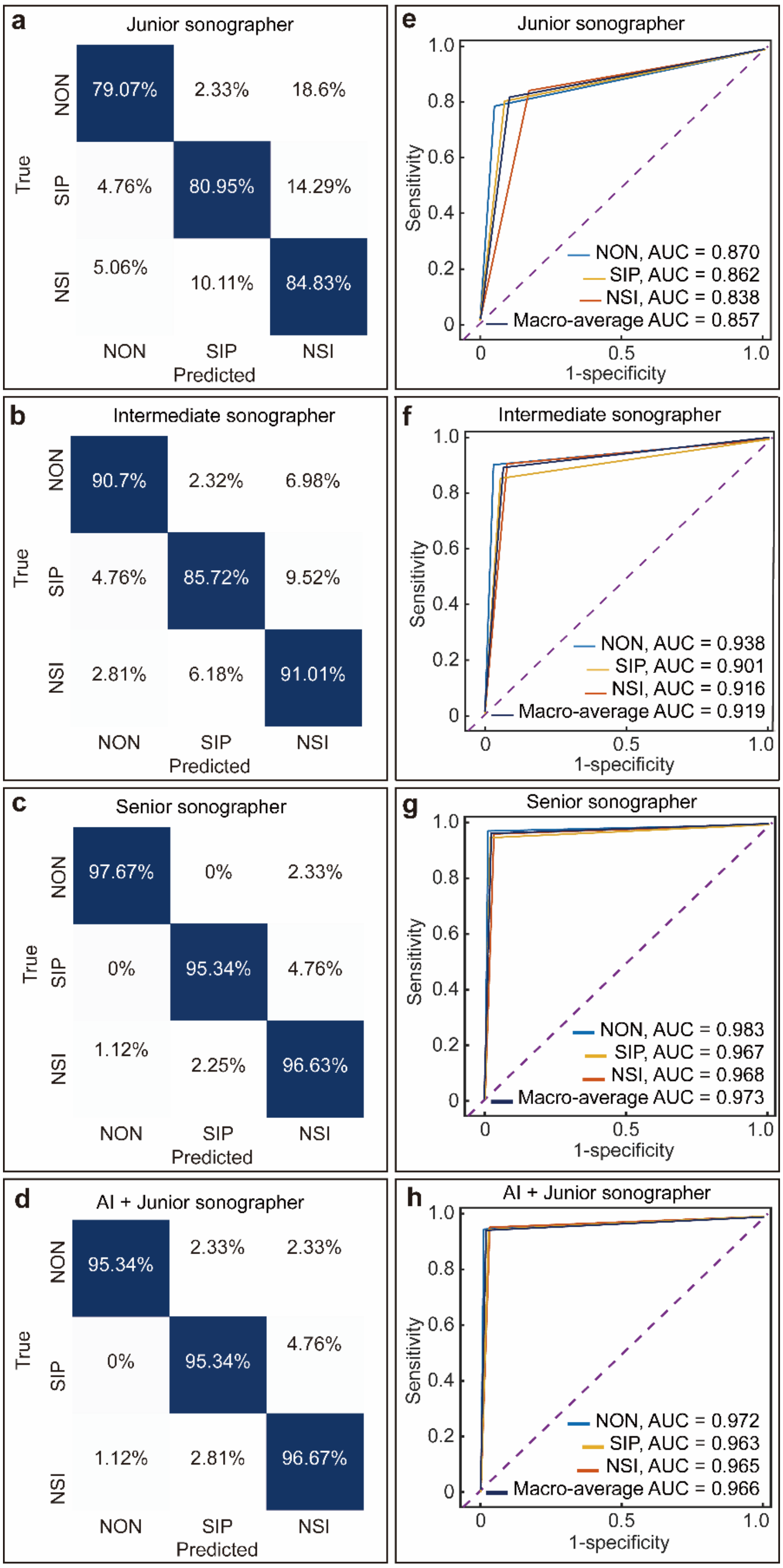

Supplemental Figure. 1 Results of four observation groups diagnosing 242 suspected ileocolic intussusceptions. **A**, **C**, **E**, and **G** the normalized confusion matrix. **B**, **D**, **F**, and **H** represent the AUC curves. NSD = Non-surgical doughnut sign. NSS = Non-surgical sleeve sign. SSI = Surgical sign.
